# Adversarial domain adaptation for false-positive reduction in hospital-to-robotic echocardiographic screening under limited target-domain data

**DOI:** 10.3389/frobt.2026.1900388

**Published:** 2026-10-21

**Authors:** Hsu Thiri Soe, Yamauchi Yuki, Hiroyasu Iwata

**Affiliations:** 1 Department of Modern Mechanical Engineering, Graduate School of Creative Science and Engineering, Waseda University, Tokyo, Japan; 2 Iwata Laboratory, Department of Modern Mechanical Engineering, Graduate School of Creative Science and Engineering, Waseda University, Tokyo, Japan

**Keywords:** DANN, domain adaptation, echocardiography, medical robotic, myocardial infarction

## Abstract

Hospital-trained deep learning models for echocardiographic wall motion abnormality detection achieve strong performance under standard clinical acquisition conditions but often degrade substantially when deployed on autonomous robotic acquisition systems. Most existing models are developed and validated under controlled clinical acquisition conditions, limiting their robustness when deployed in emerging autonomous imaging systems. A hospital-trained wall motion abnormality detection model trained on 10,000 hospital echocardiograms achieved 92.3% accuracy (94.0% sensitivity, 90.6% specificity) when applied to an independent hospital test data set of 500 studies. However, direct deployment on 150 robotic echocardiograms from 15 healthy subjects reduced the normal-case specificity to 65.7%, representing a 24.9 percentage-point drop compared to the performance when applied to the hospital-acquired data. No statistically significant difference in SNR was observed between domains (p = 0.31; d = 0.12), with similarly small standardized differences in contrast and sharpness, although formal equivalence was not established. Acquisition-geometry measures differed descriptively between domains, suggesting that geometry-associated factors may contribute to the observed domain shift. We propose an adversarial domain adaptation framework built upon a 3D ResNet-18 backbone with a gradient-reversal domain discriminator for aligning hospital and robotic feature representations while retaining the hospital-trained classification objective. This design enables adaptation to the robotic domain without requiring labeled robot-acquired pathological cases, which are particularly difficult to obtain during early-stage robotic system development. Under leave-one-subject-out validation across robotic subjects, Mixed-DANN improved specificity to 86.1% ± 4.3%, while Normal-only DANN improved specificity to 89.3% ± 4.8% (p < 0.001 versus direct transfer), recovering 94.8% of the hospital-to-robot specificity gap. Because the robotic cohort comprises only healthy subjects, evaluation is appropriately restricted to specificity and false-positive rate; sensitivity evaluation requires future acquisition of robot-acquired pathological cases. These findings suggest that acquisition-associated domain shift substantially impacts cross-platform deployment. The proposed adversarial domain adaptation framework provides a potential pathway for adapting hospital-trained cardiac AI models to autonomous robotic ultrasound under conditions of limited target-domain data, although validation using robot-acquired pathological cases remains necessary before clinical translation.

## 1 Introduction

Myocardial infarction (MI) remains a leading cause of mortality worldwide, where timely detection is essential for improving clinical outcomes (World Health Organization, 2025). Recent advances in deep learning have enabled automated analysis of echocardiographic wall motion abnormalities associated with myocardial injury, with multiple studies reporting performance approaching expert-level accuracy (Soe and Iwata, 2024; Deger et al., 2021; Liu et al., 2023). This achievement has accelerated interest in scalable, AI-assisted cardiac imaging and assessment systems (Gomez et al., 2025). In parallel, autonomous robotic echocardiography platforms have emerged as a means of reducing operator dependency and expanding access to cardiac imaging in remote and resource-limited environments. However, deploying hospital-trained echocardiographic AI models in robotic acquisition systems introduces a critical translational challenge: models trained on clinician-acquired hospital data often fail at generalization to robot-acquired images (Gotway and Liang, 2025). This failure in cross-platform deployment suggests a domain shift between hospital and robotic acquisition settings. Differences in the probe positioning, view distribution, and acquisition geometry may alter the statistical properties of the input data and learned feature representations. Domain adaptation methods have been extensively studied in computer vision and medical imaging. These methods include adversarial alignment approaches such as Domain-Adversarial Neural Networks (Bhattacharya et al., 2025), discrepancy-based methods (de Mathelin et al., 2022), and self-training strategies (Oymak and Gulcu, 2020). However, most of these methods assume moderate distributional differences or require substantial labeled target-domain data. In robotic echocardiography, target-domain data are typically scarce, particularly for pathological cases, limiting the applicability of conventional adaptation strategies. This study investigates the deployment reliability of a hospital-trained wall motion abnormality detection model when transferred from conventional hospital-acquired to robotically acquired echocardiographic data. Because only healthy robotic data are currently available, we focus specifically on normal-case specificity recovery, which is both measurable with available data and directly relevant to deployment safety, as elevated false-positive rates in autonomous acquisition systems lead to unnecessary referrals and reduced user confidence in robotic platforms. We introduce an adversarial domain adaptation framework based on gradient-reversal-based domain discrimination, which retains the hospital-trained classification objective while promoting acquisition-invariant feature representations for hospital-to-robot domain shift. Subject-level LOSO-CV demonstrates substantial recovery of robotic performance compared to direct transfer.

## 2 Related work

Deep learning has substantially advanced the automated interpretation of echocardiography data. Madani et al. presented robust, convolutional neural network (CNN)-based classification of standard echocardiographic views, demonstrating that deep architectures can learn complex spatial representations from ultrasound data (Madani et al., 2018). Zhang et al. proposed a fully automated clinical pipeline for chamber quantification and functional assessment across more than 14,000 studies, achieving expert-level performance (Zhang et al., 2018). Ouyang et al. further introduced a video-based deep learning approach for beat-to-beat cardiac function assessment, highlighting the importance of spatiotemporal modeling in echocardiography (Ouyang et al., 2020). These studies report strong diagnostic performance, but evaluations are typically conducted under homogeneous hospital acquisition conditions. Cross-platform generalization, particularly from clinician-acquired to robotic echocardiography, remains largely unexplored. Domain adaptation (DA) addresses distributional discrepancies between source and target domains. DANN introduces adversarial feature alignment via gradient reversal as a means of promoting domain-invariant representations (Ganin and Lempitsky, 2015). Discrepancy-based methods such as Maximum Mean Discrepancy minimization in Deep Adaptation Networks (Long et al., 2015) and correlation alignment (CORAL) (Sun and Saenko, 2016) reduce statistical divergence between domains without requiring extensive labeled target data. These techniques have been increasingly adopted in medical image analysis, where domain shifts commonly arise from scanner variability and cross-institutional differences. In ultrasound imaging, domain shift is particularly pronounced owing to variations in the probe orientation, operator expertise, and device configuration (Ferraz et al., 2023). Prior studies have addressed cross-scanner variability (Yap et al., 2018), cross-device compatibility (Drozdzal et al., 2018), and operator-dependent differences in image quality. However, systematic differences in the acquisition geometry, such as those introduced by robotic ultrasound systems, present distinct challenges (Ma et al., 2025). Robotic ultrasound platforms aim to standardize probe motion and reduce operator dependency, thereby enabling scalable imaging in remote or resource-limited environments (Adams et al., 2021; Jiang et al., 2023; Christiansen et al., 2025). Most prior echocardiographic deep learning studies implicitly assume that training and deployment data originate from comparable acquisition environments.

Recent advances in robotic echocardiography highlight the growing clinical relevance of autonomous cardiac imaging. Laurent et al. proposed a semi-autonomous cardiac ultrasound acquisition system integrating AI-guided robotic probe control, demonstrating the feasibility of autonomous standard view acquisition (Laurent et al., 2025). Zhang et al. developed a telerobotic system for transesophageal echocardiography incorporating hysteresis compensation to improve probe positioning accuracy (Zhang et al., 2025). These systems demonstrate that robotic acquisition introduces distinct kinematic constraints that fundamentally differ from human-operated scanning and directly motivate the need for acquisition-aware AI adaptation.

The ORIZURU seated echocardiography robot, developed at the Iwata Laboratory, Waseda University, is one example of a robotic platform for autonomous transthoracic echocardiographic acquisition. The system incorporates autonomous probe navigation and joint encoder feedback to support standardized image acquisition (Shida et al., 2023; Shida et al., 2025). In the present study, ORIZURU serves as the target robotic acquisition platform for evaluating cross-platform deployment of hospital-trained AI models. Despite growing interest in autonomous echocardiography, the specific problem of false-positive elevation under hospital-to-robot deployment and its mitigation when only healthy target-domain data are available remains insufficiently studied. The primary contribution of the present study is its application to the specific and underexplored problem of hospital-to-robot echocardiographic deployment under healthy-only target-domain data conditions, and the systematic analysis of acquisition-geometry-associated domain shift in this clinical robotics setting.

## 3 Methodology

### 3.1 Dataset description

We utilized 12,000 transthoracic echocardiographic studies acquired at a tertiary cardiac center according to the American Society of Echocardiography guidelines (Taub et al., 2025). The primary data source was the Cardiovascular Institute, Tokyo, Japan, which provided a comprehensive collection of echocardiographic images (The Cardiovascular Institute Hospital, 1959). The data collection and usage were performed with ethical approval from the Cardiovascular Institute, Japan, ensuring compliance with patient privacy regulations and research ethics guidelines. For the retrospective hospital dataset, patient-informed consent was waived by the ethics committee in accordance with institutional policy for retrospective anonymized clinical data. All methods were carried out in accordance with relevant institutional guidelines and regulations. The dataset included MI cases as well as studies of normal cardiac function. All examinations were retrospectively labeled by expert cardiologists. The hospital dataset comprised 12,000 studies in total: 10,000 studies for baseline model training (5,000 MI-positive and 5,000 normal), 500 studies for independent hospital testing (250 MI-positive and 250 normal), and 1,500 studies reserved for domain-characterization analysis (750 MI-positive and 750 normal). All partitions were defined at the patient level, and no patient overlap was permitted across the training, independent test, and domain-characterization subsets.

**Table:** 

Dataset subset	Studies	MI-positive	Normal	Purpose
Training	10,000	5,000	5,000	Baseline model training
Independent test	500	250	250	Hospital-domain evaluation
Domain characterization	1,500	750	750	Hospital–robot domain analysis
Total	12,000	6,000	6,000	-

The robotic dataset was used for target-domain adaptation and false-positive evaluation in healthy robotic echocardiograms, rather than direct validation of robotic-domain MI sensitivity.

Robotic echocardiographic acquisitions were performed on 150 view-specific echocardiographic video clips from 15 healthy volunteers using the ORIZURU robotic ultrasound acquisition platform. The ORIZURU system is a robotic ultrasound platform developed at the Iwata Laboratory, Waseda University, for transthoracic echocardiographic acquisition. The system employs a multi-degree-of-freedom robotic arm to position and orient the ultrasound probe according to predefined view-acquisition sequences targeting standard echocardiographic windows. Joint encoder data were recorded throughout each examination and were subsequently used to quantify probe-angle variability in the domain characterization analysis. All robotic examinations were performed under the supervision of an experienced cardiologist to ensure appropriate probe placement and participant safety throughout data acquisition. Additional technical details of the ORIZURU platform are available in our previous publications (Shida et al., 2023; Shida et al., 2025).

Healthy volunteers were defined as individuals with no prior history of cardiovascular disease, no current cardiac symptoms, and no known structural abnormalities, as confirmed prior to participation. All procedures performed in studies involving human participants were approved by the Ethics Review Board of Waseda University and conducted in accordance with the ethical standards of the institutional research committee in Waseda University (identification number: 2022-040). Written informed consent was obtained from all volunteer participants prior to participation. The robotic acquisitions followed standardized protocols with robotically controlled probe positioning and predetermined view sequences. Consequently, the robotic evaluation is limited to the specificity and false-positive rate. Sensitivity evaluation requires robotic-acquired MI cases and is addressed as future work.

One robotic video clip refers to a single view-specific video recording of one of three standard echocardiographic views: a parasternal long-axis (PLAX), parasternal short-axis (PSAX), and apical four-chamber (A4CH) (Figure 1) with each view recorded for approximately 2–4 s spanning multiple cardiac cycles, yielding approximately 3–4 recordings per view per subject across the 15 participants. The robotic acquisition was fully autonomous, with the Orizuru system controlling probe positioning and view acquisition without direct operator intervention during scanning. The system was developed and operated by biomedical engineering researchers under clinical supervision by an experienced cardiologist to ensure appropriate probe placement and patient safety. Video clips rather than individual frames were used as model inputs.

**FIGURE 1:**
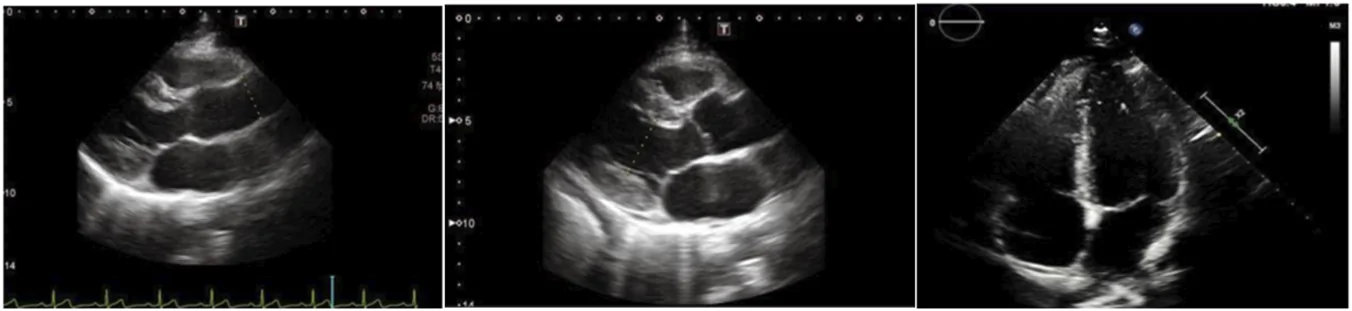
Random sample image of PLAX, PSAX, and A4CH.

The data hierarchy was defined as subject → acquisition session → echocardiographic view → video clip → video frame, with the video clip serving as the unit of model prediction and the subject serving as the unit of inferential analysis.

To ensure statistical independence, robotic-domain evaluation was conducted using leave-one-subject-out cross-validation (LOSO-CV) across the 15 robotic subjects. In each fold, one subject was held out exclusively for testing. Of the remaining 14 subjects, 12 subjects were used for adaptation training and two subjects were reserved for validation. No clips from the held-out test subject were used during training, validation, hyperparameter selection, or early stopping. Performance metrics were first calculated for each held-out subject and then summarized across the 15 LOSO-CV folds.

### 3.2 Baseline wall motion abnormality detection model

The baseline model is a 3D CNN with a ResNet-18 backbone developed for binary MI classification using spatiotemporal wall-motion information from echocardiographic videos. The ResNet-18 backbone was initialized from ImageNet-pretrained 2D weights, which were inflated to initialize the corresponding 3D convolutional kernels. The network performs binary classification (MI vs. normal). Training was performed on 10,000 hospital studies using the cross-entropy loss:

$$L_{cls}=-\frac{1}{N}\sum_{i=1}^{N}\left[y_{i}\log\left({ŷ}_{i}\right)+\left(1-y_{i}\right)\log\left(1-{ŷ}_{i}\right)\right]$$

where *y i* ∈ { 0, 1 } denotes the ground-truth label and ${ŷ}_{i}$ represents the predicted probability of MI. Standard data augmentation, including random rotation, scaling, and temporal cropping, was applied during training. When applied to the independent hospital test set comprising 500 studies, the baseline model achieved 92.3% accuracy, 94.0% sensitivity, and 90.6% specificity at the video-clip level, confirming strong in-domain diagnostic performance under standard clinical acquisition conditions. The test set comprised 1,600 video clips (800 MI-positive and 800 normal). Accuracy is reported as overall accuracy, defined as (TP + TN)/(TP + TN + FP + FN).

Because the robotic dataset contains only healthy subjects, evaluation in the robotic domain was restricted to normal-case performance. Using leave-one-subject-out cross-validation (LOSO-CV) across the 15 robotic subjects, the hospital-trained model achieved a specificity of 65.7% ± 6.4% (mean ± standard deviation across folds), corresponding to a FPR of 34.3% ± 6.4%. The hospital-trained model achieved a specificity of 90.6% on hospital-acquired normal cases; however, deployment on robotic echocardiographic acquisitions reduced specificity to 65.7%, representing a 24.9 percentage-point decrease. Sensitivity and overall diagnostic accuracy, which require MI-positive cases for computation, were therefore evaluated exclusively in the hospital domain and were not directly compared across domains.

### 3.3 Quantitative characterization of domain shift

To analyze hospital-to-robot domain differences, we compared 1,500 hospital studies and 150 robotic video clips. The signal-to-noise ratio (SNR) for the image quality metrics was computed as:

$$SNR=\frac{\mu_{cardiac}}{\sigma_{background}}$$

where $\mu_{cardiac}$ denotes the mean pixel intensity within a manually defined myocardial region of interest and $\sigma_{background}$ ​ denotes the standard deviation of pixel intensities within a background region outside the cardiac silhouette. SNR was computed per video clip and averaged at the subject level. Additional image quality metrics included myocardial-blood pool contrast, defined as the normalized intensity difference between myocardial and ventricular blood pool regions, and spatial sharpness, quantified using the variance of the Laplacian operator applied to each frame and averaged across clips per subject.

#### 3.3.1 Acquisition geometry analysis

For robotic acquisitions, probe-angle variability was quantified using joint encoder data from the Orizuru platform, computed as the standard deviation of joint-derived probe orientation angles within each view class (PLAX, PSAX, A4CH) across all frames per subject and averaged across subjects. For hospital-acquired data, direct probe-angle measurements were unavailable as clinical acquisitions do not record robotic joint encoder data. Hospital-domain geometric variability was therefore assessed indirectly through view consistency scoring, defined as the proportion of frames independently graded as diagnostically acceptable.

(Grade A or B) by two board-certified cardiologists. This approach avoids unvalidated proxy measurements while enabling descriptive characterization of geometric differences between domains. We acknowledge this asymmetry as a study limitation.

#### 3.3.2 Statistical analysis

Given the substantial difference in cohort sizes (hospital n = 1,500 vs. robotic n = 150) and population compositions (mixed MI and normal vs. healthy only), formal hypothesis testing between domains was conducted with caution. Independent-samples t-tests were applied to continuous metrics where distributional assumptions were verified using the Shapiro-Wilk test. Results are reported as mean ± standard deviation. Due to population differences, between-domain comparisons are interpreted as descriptive characterizations of acquisition-induced distributional differences rather than formal inferential claims.

Robotic-domain performance was analyzed at the subject level to account for clustering of multiple clips within each participant. Specificity and false-positive rate were first calculated independently for each held-out LOSO test subject. Across the 15 subjects, results were summarized using mean ± standard deviation and median with interquartile range (IQR). Ninety-five percent confidence intervals for mean specificity were estimated using subject-level bootstrap resampling, in which subjects rather than individual clips were resampled. Paired comparisons between direct transfer and the adapted models were assessed using the Wilcoxon signed-rank test. Pooled clip-level TN and FP counts were reported descriptively but were not treated as independent samples for subject-level inferential testing. A two-sided p value <0.05 was considered statistically significant.

#### 3.3.3 Feature space analysis

To characterize internal representation shifts, intermediate feature vectors were extracted from the penultimate layer of the baseline classification network for both hospital and robotic sequences. These features were visualized using t-distributed stochastic neighbor embedding (t-SNE) dimensionality reduction. t-SNE visualizations are treated as qualitative illustrations of feature distribution differences and are not used as standalone quantitative evidence of domain shift.

### 3.4 Adversarial domain adaptation for false-positive reduction

To mitigate cross-platform domain shift, we extend the baseline network using adversarial feature alignment. The architecture consists of a 3D ResNet-18 backbone as the feature extractor, the hospital-trained wall motion abnormality classification head, and a two-layer multilayer perceptron (MLP) domain discriminator with dimensions 512→128→1, ReLU activation after the first layer, batch normalization, dropout (p = 0.5), and sigmoid output activation. A Gradient Reversal Layer (GRL) is inserted between the feature extractor and domain discriminator. During forward propagation, the GRL acts as an identity transformation; during backpropagation, it multiplies the gradient by −λ, reversing the gradient signal to the feature extractor and thereby encouraging domain-invariant feature learning.

The classification head performs binary MI classification using echocardiographic wall-motion information and is trained exclusively on labeled hospital data. In the robotic domain, where only healthy subjects are available, adaptation targets false-positive reduction by aligning normal-case feature representations across domains without modifying the classification objective. Although the robotic target domain contains only healthy subjects, adversarial feature alignment remains applicable under the hypothesis that acquisition-associated differences contribute substantially to the observed domain shift. Because the target cohort contains only healthy subjects, the present study cannot fully disentangle acquisition effects from differences in population composition. By aligning the feature distributions of hospital and robotic normal cases, the GRL reduces acquisition-associated feature differences, thereby reducing false-positive predictions without requiring robotic MI-positive cases. This assumption cannot be directly verified without robotic MI-positive cases and is acknowledged as a limitation. However, it is supported indirectly by the domain characterization findings showing no statistically significant difference in SNR between domains (p = 0.31) and small standardized differences in other image-quality metrics, while acquisition-geometry measures differed descriptively between domains, suggesting that geometry-associated factors may contribute to the observed distributional differences.

The domain classification loss is defined as:

$$L_{domain}=-\frac{1}{N}\sum_{i=1}^{N}\left[d_{i}\log\left({\hat{d}}_{i}\right)+\left(1-d_{i}\right)\log\left(1-{\hat{d}}_{i}\right)\right]$$

where $d_{i}\in \left\{0,1\right\}$ denotes the domain label, with $d_{i}=0$ for hospital-acquired samples and $d_{i}=1$ for robot-acquired samples, and ${\hat{d}}_{i}$ denotes the domain discriminator’s predicted probability that sample i belongs to the robotic domain. The classification head weights are updated only through the supervised screening loss; the adversarial gradient from the domain discriminator is applied to the feature extractor through the GRL and does not directly update the classification head. No backbone layers were frozen during adaptation.

$$\underset{\theta_{f},\theta_{y}}{\min}L_{screening}-\lambda L_{domain}$$

The domain discriminator is trained to minimize:

$$\underset{\theta_{d}}{\min}L_{domain}$$

The adaptation coefficient was selected from λ ∈ {0.01, 0.05, 0.1, 0.2, 0.5} using a nested subject-level procedure. In each outer LOSO fold, one robotic subject was held out exclusively for final testing. Among the remaining 14 robotic subjects, 12 were used for adaptation training and two were used for inner validation. The value of λ was selected based on normal-case specificity calculated across the two robotic inner-validation subjects. Hospital-domain classification validation loss was monitored separately for early stopping. The held-out outer-test subject and the independent 500-study hospital test set were not used for hyperparameter selection, checkpoint selection, early stopping, or threshold determination. Based on the inner-validation analysis, λ = 0.1 was selected before final evaluation.

Within each outer LOSO fold, a patient-level validation subset drawn exclusively from the 10,000-study hospital training partition was used to monitor the hospital-domain classification loss for early stopping. The independent 500-study hospital test set remained completely untouched during training, hyperparameter selection, early stopping, and model selection, and was used only for final pre- and post-adaptation evaluation. The screening loss is computed using labeled hospital data only. The domain loss is computed using both hospital- and robot-acquired samples. Figure 2 illustrates the proposed framework architecture.

**FIGURE 2:**
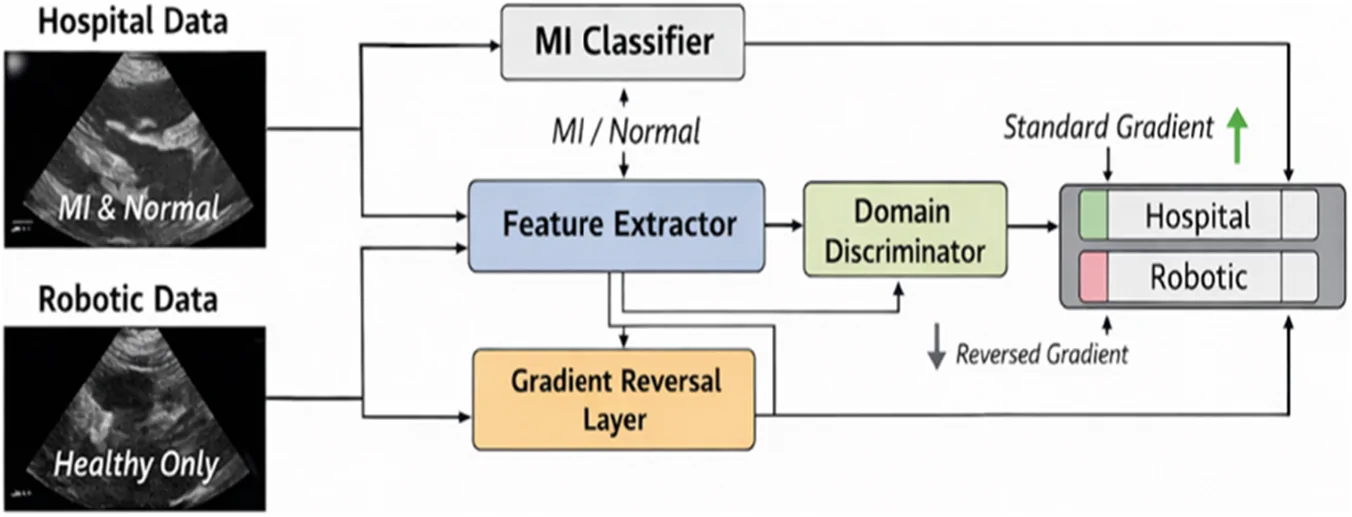
The proposed domain adaptation framework.

Two domain-adaptation configurations were evaluated. In the Mixed-DANN configuration, both MI-positive and normal hospital samples contributed to the domain-adversarial loss together with healthy robot-acquired samples. In the Normal-only DANN configuration, the supervised classification loss continued to use both MI-positive and normal hospital samples, whereas the domain-adversarial loss was restricted to normal hospital samples and healthy robot-acquired samples. This additional configuration was introduced to reduce potential confounding arising from the difference in disease-status composition between the mixed hospital source domain and the healthy-only robotic target domain.

#### 3.4.1 Training details

Subject-level LOSO-CV was employed across the 15 robotic subjects. In each fold, one robotic subject was held out exclusively for testing, while 12 of the remaining subjects were used for adaptation training and two were reserved for validation. The held-out test subject was not used for model selection, hyperparameter selection, or early stopping.

#### 3.4.2 Evaluation metrics

Performance on robot-acquired data was evaluated using normal-case specificity, defined as TN/(TN + FP), and false-positive rate. Each view-specific robotic video clip produced one binary prediction using a fixed classification threshold of 0.50. A healthy robotic clip predicted as normal was counted as a true negative, whereas a healthy robotic clip predicted as MI-positive was counted as a false positive. Clip-level predictions were aggregated within each held-out subject to calculate subject-level specificity and FPR. Hospital-domain performance was evaluated using accuracy, sensitivity, specificity, and area under the receiver operating characteristic curve (AUROC). Because the robotic cohort contained no MI-positive cases, robotic-domain sensitivity and overall diagnostic accuracy were not estimated. Pre-adaptation and post-adaptation hospital-domain performance was evaluated at the video-clip level on the same independent hospital test set comprising 500 studies and 1,600 video clips (800 MI-positive and 800 normal), using the same fixed classification threshold of 0.50 and the same preprocessing pipeline. Exact clip-level confusion matrices were calculated for each model, and descriptive 95% Wilson binomial confidence intervals for accuracy, sensitivity, and specificity were estimated at the clip level.

## 4 Experiments

Because native frame rates varied across acquisitions, echocardiography video clips were temporally standardized by uniformly sampling 30 frames over each clip rather than by resampling to a fixed frame rate. Each frame was processed as a single-channel grayscale image and resized to 256 × 256 pixels. Pixel intensities were normalized using the mean and standard deviation computed from the hospital training set. The same preprocessing procedure was applied identically at test time. Data augmentation included random rotation (±10°), scaling (0.9–1.1×), and temporal cropping. Optimization was performed using stochastic gradient descent, learning rate: 0.001, momentum: 0.9, weight decay: 1 × 10^−4^ for 100 epochs with a batch size of 8. During adversarial adaptation, each mini-batch contained four hospital-domain samples and four robot-domain samples. Hospital-domain samples were randomly drawn from the source dataset without enforcing additional within-batch MI/normal class balancing. For adversarial adaptation, the gradient reversal coefficient was set to λ = 0.1. Models were trained for a maximum of 100 epochs. Hospital-domain validation loss was monitored after each epoch, and the checkpoint with the lowest validation loss was retained for evaluation. No patience-based early-stopping rule was applied.

Subject-level LOSO-CV was conducted across 15 robot-acquired subjects. In each fold, one subject was held out for testing, while the remaining 14 subjects were divided into 12 subjects for adaptation training and two subjects for validation. Performance is reported as mean ± standard deviation across 15 folds. Random seeds were fixed to improve reproducibility. Hyperparameter candidate values were prespecified, and the gradient-reversal coefficient was selected exclusively using the inner-validation procedure described above.

## 5 Results

### 5.1 Baseline performance of model applied to hospital-acquired data

The baseline 3D ResNet-18 wall motion abnormality detection model was first evaluated on the independent hospital test set (n = 500) containing both MI-positive and MI-negative cases. The model achieved 92.3% overall accuracy, 94.0% sensitivity, and 90.6% specificity, confirming strong in-domain diagnostic performance under standard clinical acquisition conditions. Overall accuracy is defined as (TP + TN)/(TP + TN + FP + FN).

### 5.2 Cross-domain deployment

We evaluated the hospital-trained model by applying it to 150 robotic echocardiographic clips from 15 pathology-negative healthy subjects using subject-level LOSO-CV. Because the robot-acquired dataset contains only healthy subjects, cross-domain evaluation was restricted to normal-case specificity and false-positive rate (FPR). Metrics requiring MI-positive cases, including sensitivity and overall accuracy, were therefore evaluated only in the hospital domain and were not compared across domains.

Under direct-transfer conditions (no adaptation), normal-case specificity decreased from 90.6% on hospital-acquired normal cases to 65.7% ± 6.4% (95% CI: 62.6%–68.8%; median 66.7%, IQR 60.0%–70.0%) on robotic acquisitions, corresponding to an FPR of 34.3% ± 6.4%. This represents a 24.9 percentage-point reduction in normal-case specificity, indicating substantial degradation following deployment across acquisition platforms.

Per-subject specificity results under direct-transfer conditions are summarized in Table 1 Subject-level specificity ranged from 55.6% to 75.0%, with all subjects falling below the hospital-domain normal-case specificity of 90.6%, confirming consistent cross-platform performance degradation across the entire robotic cohort.

**TABLE 1:** Per-subject normal-case specificity before and after adversarial domain adaptation. The 95% confidence intervals for mean specificity were estimated using subject-level bootstrap resampling.

Subject	Clips	Baseline	Mixed-DANN	Normal-only DANN
R01	11	63.6%	81.8%	90.9%
R02	9	66.7%	88.9%	88.9%
R03	10	70.0%	90.0%	90.0%
R04	12	58.3%	83.3%	91.7%
R05	8	75.0%	87.5%	100.0%
R06	10	60.0%	80.0%	90.0%
R07	11	72.7%	90.9%	90.9%
R08	9	55.6%	77.8%	77.8%
R09	10	70.0%	90.0%	90.0%
R10	12	58.3%	83.3%	91.7%
R11	8	75.0%	87.5%	87.5%
R12	10	60.0%	90.0%	90.0%
R13	11	63.6%	81.8%	81.8%
R14	9	66.7%	88.9%	88.9%
R15	10	70.0%	90.0%	90.0%
Mean ± SD		65.7% ± 6.4%	86.1% ± 4.3%	89.3% ± 4.8%
95% CI		62.6%–68.8%	83.9%–88.1%	86.9%–91.6%
Median [IQR]		66.7% [60.0–70.0]	87.5% [82.6–90.0]	90.0% [88.9–90.9]

### 5.3 Quantitative characterization of domain shift

To investigate factors associated with the hospital-to-robot performance gap, image quality and acquisition geometry were compared using 1,500 hospital studies and 150 robotic video clips.

SNR showed no significant difference (p = 0.31), with a standardized effect size of d = 0.12, indicating negligible difference in global image quality. Standardized differences were also small for myocardial-blood pool contrast (d = 0.09) and spatial sharpness (d = 0.11). We note that non-significant p-values do not establish equivalence; the small standardized effect sizes provide descriptive support for comparable image quality across domains, but formal equivalence testing was not conducted.

Acquisition geometry was characterized separately in each domain due to measurement asymmetry. In the robotic domain, probe-angle variability was quantified as the standard deviation of joint-encoder-derived orientation angles, yielding a mean variability of 12.3° ± 3.1° across subjects. In the hospital domain, direct probe-angle measurements were unavailable; geometric variability was instead characterized by expert-rated view consistency, with 95.2% ± 3.1% of hospital frames rated diagnostically acceptable compared with 60.3% ± 8.7% in robotic acquisitions. These two measures are not directly comparable and are reported as descriptive indicators of geometric differences between domains rather than quantities supporting a formal cross-domain statistical test. This measurement asymmetry is acknowledged as a study limitation.

A sensitivity analysis restricted to normal hospital cases only (n = 750) confirmed that the pattern of results was consistent with the full-cohort analysis. View-consistency scores remained substantially lower in robotic acquisitions (60.3% ± 8.7%) compared with normal hospital cases only (94.8% ± 3.4%), indicating that the observed pattern persisted after restricting the hospital comparison to normal cases.

To provide quantitative evidence of domain-invariant feature alignment, domain discriminator performance was evaluated before and after adaptation (Table 2). Before adaptation, the discriminator achieved 92.0% accuracy (AUROC = 0.95), confirming a substantial and learnable domain gap. After Mixed-DANN adaptation, discriminator accuracy decreased to 56.0% (AUROC = 0.57), and after Normal-only DANN adaptation to 53.5% (AUROC = 0.54), both approaching chance level (50.0%, AUROC = 0.50). These results provide quantitative evidence of reduced domain separability after adaptation, complementing the qualitative t-SNE visualization.

**TABLE 2:** Quantitative domain discriminator performance before and after adaptation.

Stage	Discriminator accuracy	Discriminator AUROC
Before adaptation	92.0%	0.95
After Mixed-DANN	56.0%	0.57
After normal-only DANN	53.5%	0.54
Chance level	50.0%	0.50

t-SNE visualization (perplexity = 30, learning rate = 200, random seed = 42; n = 150 robotic and 1,500 hospital feature vectors) showed clear separation between hospital and robotic feature clusters prior to adaptation (Figure 3). Following adaptation, increased overlap between feature clusters was observed, consistent with the quantitative discriminator results. This visualization is illustrative and should not be interpreted inferentially.

**FIGURE 3:**
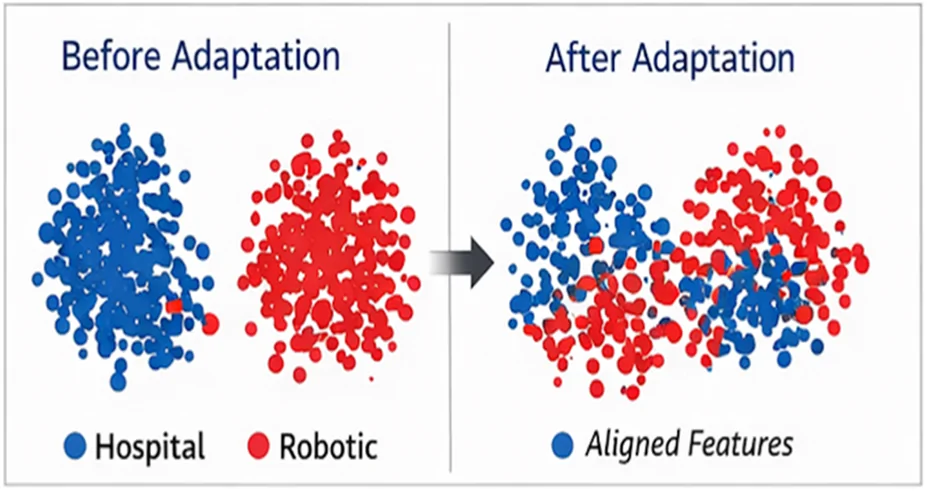
Qualitative t-SNE visualization of hospital and robotic feature embeddings before and after adversarial adaptation. The visualization includes 1,500 hospital and 150 robotic feature vectors and was generated using a perplexity of 30, a learning rate of 200, and random seed 42. The visualization is illustrative and is not used as inferential evidence.

These findings collectively suggest that the hospital-to-robot domain shift is associated with acquisition-geometry differences rather than conventional image-quality degradation, although direct causal attribution is not possible under the current study design.

### 5.4 Effect of adversarial domain adaptation

Two adversarial domain adaptation strategies were evaluated: Mixed-DANN, using all hospital samples for the domain loss, and Normal-only DANN, restricting domain loss computation to normal hospital samples only. Both strategies retained MI-positive and normal cases for the classification loss.

Under subject-level LOSO-CV across 15 robotic subjects, both strategies substantially improved normal-case specificity compared with direct transfer. Under direct-transfer conditions, robotic-domain specificity was 65.7% ± 6.4% (95% CI: 62.6%–68.8%). Following Mixed-DANN adaptation, specificity increased to 86.1% ± 4.3% (95% CI: 83.9%–88.1%), and following Normal-only DANN adaptation to 89.3% ± 4.8% (95% CI: 86.9%–91.6%). Wilcoxon signed-rank tests confirmed that both improvements were statistically significant (p < 0.001 for both comparisons), with a rank-biserial correlation of r = 1.0 for both Mixed-DANN and Normal-only DANN. All 15 subjects showed improved specificity under both adaptation strategies relative to direct transfer.

Normal-only DANN yielded numerically higher robotic-domain specificity than Mixed-DANN (89.3% vs. 86.1%), consistent with the possibility that restricting domain alignment to normal cases may reduce class-related confounding in the domain discriminator.

Per-view specificity results revealed an informative pattern consistent with the acquisition-geometry hypothesis as follows:

**Table:** 

View	Clips	Baseline specificity	Mixed-DANN	Normal-only DANN
PLAX	46	71.7%	100.0%	100.0%
PSAX	49	65.3%	100.0%	100.0%
A4CH	55	60.0%	61.8%	70.9%

PLAX and PSAX views achieved perfect specificity after adaptation under both strategies. These per-view specificity values represent pooled clip-level performance across the robotic cohort and should not be interpreted as indicating perfect performance for every individual subject. The A4CH view showed the lowest baseline specificity and retained the greatest residual false-positive burden after adaptation.

To assess whether adaptation preserved hospital-domain diagnostic performance, the adapted models were evaluated at the video-clip level on the independent 500-study hospital test set comprising 1,600 video clips (800 MI-positive and 800 normal), using the same fixed threshold (0.50) (Table 3).

**TABLE 3:** Post-adaptation hospital-domain diagnostic performance evaluated at the video-clip level on the independent 500-study hospital test set (1,600 clips) at a fixed threshold of 0.50. Values in parentheses indicate 95% Wilson binomial confidence intervals.

Metric	Baseline	Mixed-DANN	Normal-only DANN
Accuracy	92.3% (90.9–93.5)	89.9% (88.3–91.3)	91.1% (89.6–92.4)
Sensitivity	94.0% (92.1–95.4)	92.3% (90.2–93.9)	93.3% (91.3–94.8)
Specificity	90.6% (88.4–92.5)	87.5% (85.0–89.6)	89.0% (86.6–91.0)
AUROC	0.961	0.948	0.955

At the fixed threshold of 0.50, the clip-level confusion matrices were TP = 752, FN = 48, TN = 725, and FP = 75 for the baseline model; TP = 738, FN = 62, TN = 700, and FP = 100 for Mixed-DANN; and TP = 746, FN = 54, TN = 712, and FP = 88 for Normal-only DANN. Post-adaptation evaluation showed a modest reduction in hospital-domain performance. Compared with the baseline model, Mixed-DANN reduced accuracy, sensitivity, and specificity by 2.4, 1.7, and 3.1 percentage points, respectively, whereas Normal-only DANN reduced these metrics by 1.2, 0.7, and 1.6 percentage points, respectively. AUROC decreased from 0.961 at baseline to 0.948 with Mixed-DANN and 0.955 with Normal-only DANN. Thus, Normal-only DANN showed better preservation of hospital-domain diagnostic performance while substantially improving specificity in the healthy robotic target domain.

## 6 Discussion

The results indicate that hospital-trained echocardiographic AI models for wall motion abnormality detection experience substantial performance degradation when deployed on autonomous robotic acquisition systems. Although the baseline model achieved strong in-domain performance on hospital-acquired data (92.3% accuracy, 94.0% sensitivity, and 90.6% specificity), direct deployment to robotic acquisitions reduced normal-case specificity to 65.7%. Notably, this degradation was observed despite the robotic cohort consisting entirely of healthy subjects, suggesting that the observed performance gap was associated with acquisition-related domain differences rather than pathological variation.

Quantitative characterization of domain shift provided further insight into the underlying causes of this degradation. No statistically significant difference in SNR was observed between hospital and robotic acquisitions. Standardized differences in myocardial-blood pool contrast and spatial sharpness were also small. However, formal equivalence was not established. In contrast, probe-positioning variability and view-consistency measures revealed substantial acquisition-geometry differences between domains. These findings suggest that the hospital-to-robot domain shift may be substantially associated with acquisition geometry and its resulting influence on learned feature representations, although this interpretation is based on indirect evidence rather than direct causal verification. Feature-space analysis further supported this interpretation, with clear separation between hospital and robotic feature distributions observed prior to adaptation.

Post-adaptation evaluation on the independent 500-case hospital test set showed that adversarial alignment resulted in only a modest reduction in hospital-domain diagnostic performance. Under Normal-only DANN, sensitivity decreased by 0.7 percentage points (94.0% → 93.3%) and specificity by 1.6 percentage points (90.6% → 89.0%) relative to the baseline model, indicating a modest source-domain performance trade-off following adaptation.

The ablation comparing Mixed-DANN and Normal-only DANN strategies provides additional methodological insight. Restricting domain loss computation to normal hospital samples rather than all hospital samples including MI-positive cases yielded higher robotic-domain specificity (89.3% vs. 86.1%) and better preservation of hospital-domain performance. This finding is consistent with the possibility that including MI-positive cases in the domain discriminator may introduce class-related confounding, and suggests that normal-only alignment may be advantageous when the target domain contains only healthy subjects.

Per-view analysis revealed that PLAX and PSAX views achieved perfect specificity (100%) after adaptation under both strategies, while the A4CH view showed the most residual false positives (70.9% under Normal-only DANN). This pattern indicates that the A4CH view remained the most challenging view with respect to residual false-positive predictions after adaptation.

Compared with direct transfer, Normal-only DANN adversarial alignment reduced the false-positive rate from 34.3% to 10.7%, corresponding to a reduction of 23.6 percentage points. Specificity improved from 65.7% to 89.3%, recovering 94.8% of the hospital-to-robot specificity gap. Wilcoxon signed-rank testing confirmed that this improvement was statistically significant (p < 0.001), with all 15 subjects showing improvement. Hospital-domain confidence intervals were estimated at the clip level; because the original study-to-clip mapping was not retained for this analysis, within-study clustering could not be incorporated into these confidence intervals.

Comparison with prior domain adaptation literature suggests that acquisition-associated geometric domain shifts may represent a particularly suitable target for adversarial feature alignment. Unlike many medical imaging adaptation scenarios involving scanner, institution, or pathology-driven distributional differences, the hospital-to-robot shift observed in this study appears consistent with acquisition-geometry differences. Although direct comparison with alternative domain adaptation frameworks, including CORAL (Sun and Saenko, 2016) and MMD-based methods (Long et al., 2015), would be valuable, such a benchmark was beyond the scope of the present study, whose primary objective was to investigate whether adversarial feature alignment can mitigate hospital-to-robot deployment degradation under limited target-domain data conditions. Future work will include systematic comparison with alternative adaptation strategies on larger robotic datasets to establish their relative effectiveness.

These findings have potential implications for the clinical deployment of robotic cardiac AI systems. The 34.3% false-positive rate observed under direct-transfer conditions raises substantial concerns regarding autonomous robotic deployment of the hospital-trained model without continuous human oversight. The proposed adaptation framework reduced the false-positive rate to 10.7% under Normal-only DANN, substantially improving deployment reliability and supporting further prospective investigation, provided that appropriate safeguards and human review protocols remain in place. The present study addresses a specific deployment reliability problem that can be evaluated using the available robotic dataset. Because only healthy robotic participants were available, evaluation is appropriately restricted to specificity and false-positive rate. Sensitivity is an essential metric for complete clinical validation of any diagnostic system and must be evaluated using robot-acquired pathological cases before any clinical deployment. Consequently, prospective evaluation using larger and clinically diverse robotic cohorts remains essential.

Despite these limitations, the present study demonstrates that strong hospital-domain performance alone is insufficient to ensure robust deployment of cardiac AI systems in robotic acquisition environments. Acquisition-associated domain shift can substantially alter learned feature distributions and lead to clinically significant increases in false-positive rates. By explicitly addressing these shifts through adversarial feature alignment, the proposed framework provides a practical approach for improving the robustness of hospital-trained echocardiographic AI models under limited target-domain data conditions. More broadly, these findings highlight the potential importance of acquisition-aware adaptation strategies for cross-platform deployment of medical AI systems beyond robotic echocardiography, including handheld ultrasound and point-of-care imaging applications.

## 7 Limitations

Several limitations should be acknowledged. First, the robot-acquired dataset consists exclusively of pathology-negative healthy subjects, reflecting practical constraints in the early-stage development of robot-based systems. Consequently, the robotic-domain sensitivity could not be evaluated and robotic-domain evaluation was restricted to normal-case specificity and false-positive rate. Validation using robot-acquired cases with wall motion abnormalities is necessary before clinical deployment.

Second, the robot-acquired cohort includes only 15 subjects. This limits statistical power and generalizability. Although LOSO-CV was used to maximize data utilization and prevent data leakage across all available subjects, and the improvement from adversarial adaptation was confirmed statistically significant by Wilcoxon signed-rank testing (p < 0.001 for Normal-only DANN), larger multi-center robot-acquired datasets are required to confirm the generalizability of the findings.

Third, the main parameter examined for domain characterization is geometric variability in the robotic domain, quantified from joint encoder data. Direct probe-angle measurements were unavailable for the hospital domain; geometric variability was instead characterized indirectly through expert-rated view consistency. These two measures are not directly comparable.

The evidence supports an association between robotic acquisition and lower view consistency but does not constitute a direct quantitative comparison of probe-angle variability across domains. Although the results suggest that acquisition-geometry differences contribute substantially to the observed domain shift, additional factors may also contribute, including device-specific image formation characteristics.

Fourth, the robotic acquisition protocol captures three standard echocardiographic views (PLAX, PSAX, and A4CH) but does not include the Apical 2-Chamber view, which is important for comprehensive wall motion assessment under the ASE 17-segment model. The current protocol was designed around the views consistently and reliably obtainable during this initial robotic data collection study; future work should investigate reliable robotic A2CH acquisition to improve anatomical coverage.

Fifth, the hospital and robotic datasets differ simultaneously in both population composition and acquisition modality, limiting clear attribution of the observed domain shift to acquisition geometry alone. A sensitivity analysis restricted to normal hospital cases (n = 750) confirmed that the pattern of results was consistent with the full-cohort analysis; however, this does not fully eliminate the confound. Future studies should incorporate matched acquisition conditions such as robotic acquisition in hospital patients or handheld acquisition in healthy volunteers to better isolate the contribution of acquisition modality to cross-domain performance degradation.

Finally, the present study evaluated two domain alignment strategies, Mixed-DANN and Normal-only DANN, but did not systematically compare against other established domain adaptation methods such as CORAL or MMD-based approaches. Such comparison on a larger robotic cohort is identified as an important direction for future work.

## 8 Conclusion

This study investigates the deployment reliability of hospital-trained echocardiographic AI models when transferred from conventional hospital acquisition to autonomous robotic echocardiography. The present work does not constitute full clinical validation because only healthy robot-acquired data were available. The results indicate that hospital-trained echocardiographic AI models for wall motion abnormality detection experience significant cross-platform performance degradation when deployed on autonomous robotic echocardiography systems, even in the absence of pathological variation. Quantitative analysis suggests that acquisition-geometry differences, rather than conventional image-quality degradation, may contribute substantially to the observed domain shift. To address this challenge, we proposed an adversarial domain adaptation framework built upon a 3D ResNet-18 backbone for spatiotemporal echocardiographic feature extraction. By incorporating a gradient-reversal-based domain discriminator while adapting the learned feature representation through adversarial domain alignment, the framework promotes acquisition-invariant feature representations without requiring retraining of the diagnostic classifier from scratch. Under limited target-domain data conditions, the proposed Normal-only DANN approach substantially reduced false positives in robot-acquired echocardiographic data, recovering 94.8% of the hospital-to-robot specificity gap while preserving hospital-domain sensitivity to within 0.7 percentage points of the baseline model.

These findings indicate that strong in-domain performance alone is insufficient to ensure reliable normal-case performance under acquisition-domain shifts and emphasize the potential value of acquisition-aware adaptation for reducing false positives in cross-platform medical AI deployment. More broadly, this work supports further investigation of acquisition-aware adaptation for hospital-trained cardiac AI models deployed on emerging robotic ultrasound platforms and other non-standard acquisition environments.

## Data Availability

The raw data supporting the conclusions of this article will be made available by the authors, without undue reservation.
